# Disease Ontology 2015 update: an expanded and updated database of human diseases for linking biomedical knowledge through disease data

**DOI:** 10.1093/nar/gku1011

**Published:** 2014-10-27

**Authors:** Warren A. Kibbe, Cesar Arze, Victor Felix, Elvira Mitraka, Evan Bolton, Gang Fu, Christopher J. Mungall, Janos X. Binder, James Malone, Drashtti Vasant, Helen Parkinson, Lynn M. Schriml

**Affiliations:** 1 Center for Biomedical Informatics and Information Technology, National Cancer Institute, 9609 Medical Center Drive, Rockville, MD 20850, USA; 2 Institute for Genome Sciences, University of Maryland School of Medicine, Baltimore, MD 21201, USA; 3 PubChem, National Center for Biotechnology Information, National Library of Medicine National Institutes of Health Department of Health and Human Services 8600 Rockville Pike, Bethesda, MD 20894, USA; 4 Genomics Division, Lawrence Berkeley National Laboratory, Berkeley, CA 94720, USA; 5 Structural and Computational Biology Unit, European Molecular Biology Laboratory (EMBL), Heidelberg, 69117, Germany; 6 Bioinformatics Core Facility, Luxembourg Centre for Systems Biomedicine (LCSB), University of Luxembourg, Esch-sur-Alzette, 4362, Luxembourg; 7 European Molecular Biology Laboratory, European Bioinformatics Institute (EMBL-EBI), Wellcome Trust Genome Campus, Hinxton, Cambridge CB10 1SD, UK; 8 Department of Epidemiology and Public Health, University of Maryland School of Medicine, Baltimore, MD 21201, USA

## Abstract

The current version of the Human Disease Ontology (DO) (http://www.disease-ontology.org) database expands the utility of the ontology for the examination and comparison of genetic variation, phenotype, protein, drug and epitope data through the lens of human disease. DO is a biomedical resource of standardized common and rare disease concepts with stable identifiers organized by disease etiology. The content of DO has had 192 revisions since 2012, including the addition of 760 terms. Thirty-two percent of all terms now include definitions. DO has expanded the number and diversity of research communities and community members by 50+ during the past two years. These community members actively submit term requests, coordinate biomedical resource disease representation and provide expert curation guidance. Since the DO 2012 NAR paper, there have been hundreds of term requests and a steady increase in the number of DO listserv members, twitter followers and DO website usage. DO is moving to a multi-editor model utilizing Protégé to curate DO in web ontology language. This will enable closer collaboration with the Human Phenotype Ontology, EBI's Ontology Working Group, Mouse Genome Informatics and the Monarch Initiative among others, and enhance DO's current asserted view and multiple inferred views through reasoning.

## INTRODUCTION

Human disease data is a cornerstone of biomedical research for identifying drug targets, connecting genetic variations to phenotypes, understanding molecular pathways relevant to novel treatments and coupling clinical care and biomedical research (1,2). Consequently, across the multitude of biomedical resources there is a significant need for a standardized representation of human disease to map disease concepts across resources, to connect gene variation to phenotypes and drug targets and to support development of computational tools that will enable robust data analysis and integration (3,4). Defining a biomedical domain within the context of an ontology creates a rigorous knowledge backbone for the annotation of biomedical data through defined concepts connected by specified relations (5,6). Ontologies with their clearly-defined and well-structured descriptions are vital tools for the effective application of ‘omic' information through computational approaches.

**Figure 1.**
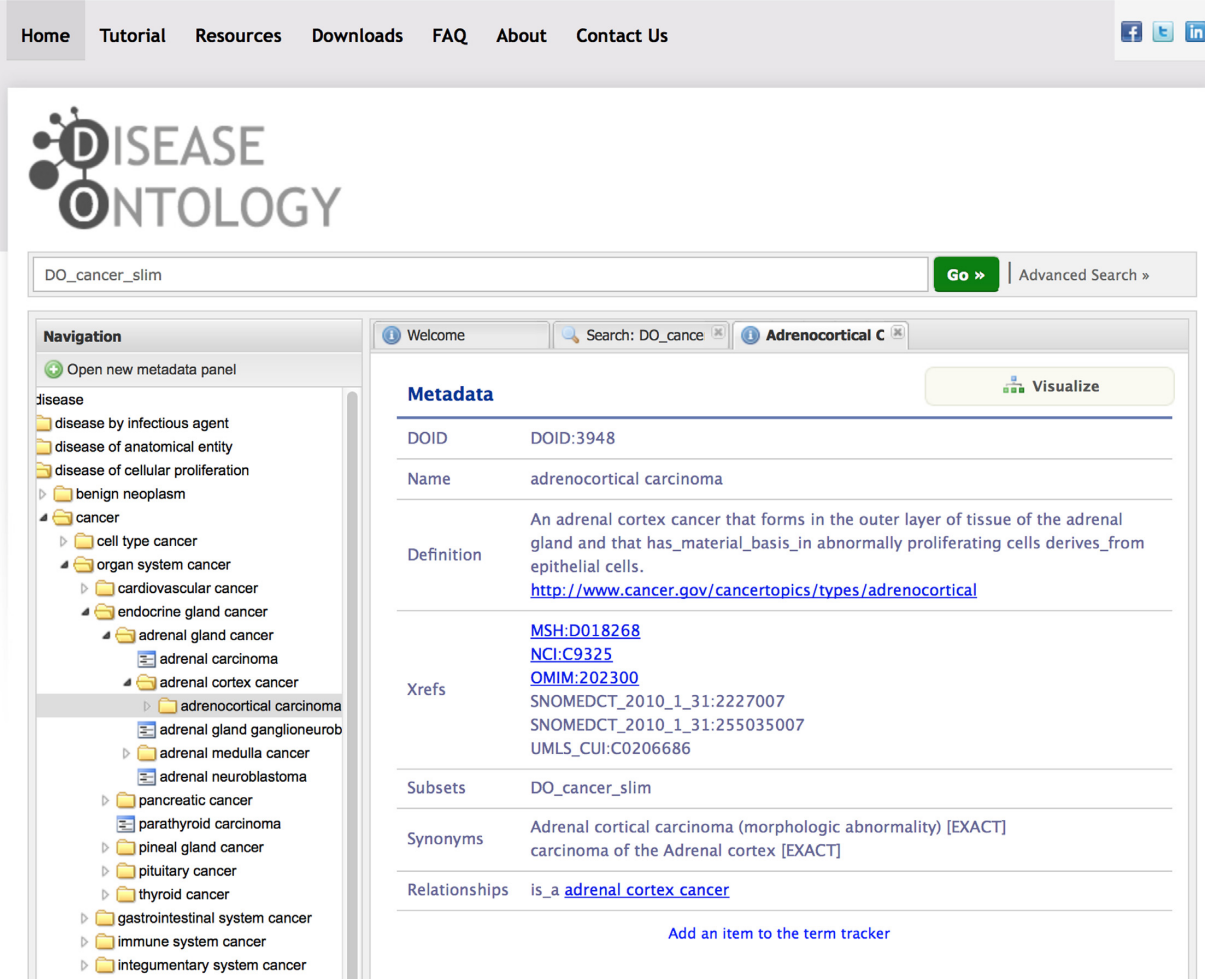
The DO website. The query for all DO terms included in the DO Cancer slim is displayed.

The Human Disease Ontology (Figure 1) (7) (DO, http://www.disease-ontology.org) is a community driven standards-based ontology that is focused on representing common and rare disease concepts captured across biomedical resources with the mission of providing a disease interface between data resources through ongoing support (term review and integration) of disease terminology needs. The DO project has had a significant impact on the development of biomedical resources, as evidenced by the body of 95 Google Scholar citations to DO's 2012 NAR paper (7).

The Human DO includes only concepts of disease and by design is meant to be a disease-focused scaffold for associating additional facts about disease. DO does not include progression (early, late, metastasis, stages) or manifestations (transient, acute, chronic) of disease as part of the definition of disease. DO does not intentionally include compound disease terms (those describing the combination of two disease terms) such as glaucoma associated with pupillary block, rather these diseases are represented by two distinct disease terms. DO integrates disease concepts from ICD-9, the National Cancer Institute (NCI) Thesaurus (8), SNOMED-CT (10) and MeSH (https://www.nlm.nih.gov/mesh/MBrowser.html) extracted from the Unified Medical Language System (UMLS) (9) based on the UMLS Concept Unique Identifiers for each disease term. DO also includes disease terms extracted directly from Online Mendelian Inheritance in Man (OMIM) (11), the Experimental Factor Ontology (EFO, http://www.ebi.ac.uk/efo/) and Orphanet (12).

## THE ENHANCED HUMAN DO

In this submission, we report on major enhancements to the DO database since 2012 including content growth, improved data structure, new areas of community based curation efforts, new community partners and a switch to web ontology language (OWL) based curation of DO. The DO website has been maintained with periodic data updates. Improvement of data content has been the primary focus of the past two years. These updates further expand the utility of DO for representing common and rare human diseases, assessment of genomic variants among human cancers, defining human disease within model organism databases, unifying disparate disease representations, connecting phenotypes to disease and connecting Big Data knowledge between pathway, protein and drug target databases.

### DO content update

We report here the expanded and more highly curated content of the DO. The DO team has committed 192 revisions to the HumanDO.obo file since our NAR 2012 paper. The latest revision of DO [revision 2702, 6 October 2014] with 8803 classes (terms) [6419 non-obsolete, 2384 obsolete] represents an increase of 760 terms since DO's previous NAR database update (8043 terms, (7)). The DO has increased the number of textual definitions from 22 to 32% of DO terms defined (2087/6419).

During this time DO's curatorial efforts have focused on enriching the content of rare genetic diseases, cardiovascular diseases, neurodegenerative diseases, inherited metabolic disorders, diseases related to intellectual disabilities and cancer. The focus of these curation efforts has been driven by requests from the research community for new terms and by requests for review of groups of terms, review of areas of DO or sets of disease terms being utilized within a biomedical resource.

### Rare genetic diseases in DO

Since the 2012 NAR paper, we have improved DO's representation of genetic disease and rare diseases in collaboration with OMIM, Orphanet (http://www.orpha.net) and National Organization for Rare Disorders (https://www.rarediseases.org). These resources provide rich clinical descriptions of rare disease prevalence, inheritance and epidemiology for the set of ∼6800 rare diseases. To date, DO has not distinguished between rare and common disease terms. In future revisions, DO will integrate disease prevalence to augment DO's disease classification. Disease prevalence, defined as the proportion of a population that is affected, can then be utilized to designate rare diseases in DO and harmonize with the European (1 in 2000 affected persons) and USA (fewer than 200 000 affected individuals) definitions of rare.

### DO structure updates

We report here examples of the structural improvements in the third and fourth tier of DO. Since the previous DO NAR report (7), the subtypes of each body system disease have been reviewed and classified. For example, to update the classification of cardiovascular system disease terms, Harrison's Principles of Internal Medicine (13) was utilized to more specifically categorize disease terms into four types of cardiovascular disease: heart conduction disease, heart disease, pericardium disease and vascular disease. All DO terms under the cardiovascular system disease node were reviewed, their parentage was assessed and updated as needed and additional nodes were defined as subtype categories, for example atrioventricular block and sinoatrial node disease were added as subtypes of heart conduction disease.

The structure of the genetic disease parent node in DO has been restructured. Genetic diseases in DO are subtyped into chromosomal disease or monogenic disease. There are currently seven DO terms that do not fit into either of these two subtypes. These diseases are subtyped directly under genetic disease as their etiology involves causal mutations within an unknown pattern of inheritance, multi-genic mutations or multiple patterns of inheritance (e.g. Coffin-Siris syndrome).

### Automated DO to OMIM mapping pipeline

In this report, we describe the results of an automated OMIM to DO mapping pipeline devised to identify candidate mappings. The pipeline was developed as part of the DISEASES (Disease-gene associations mined from literature, http://diseases.jensenlab.org/Search) project. A dictionary of DO terms and synonyms were matched to titles and acronyms from OMIM disease pages (14). Filtering rules were applied to achieve a one-to-one OMIM to DO mapping to eliminate false matches between similarly named but distinct OMIM titles. The filtering rules included: removal of possessive case (e.g. Alzheimer's), stripping punctuations, quotes and parenthesis, removal of various prefixes and postfixes and other ontology specific words (e.g. finding, Ambiguous) and changing Roman numbers to Arabic numbers. Between November 2013 and April 2014 this collaboration produced 658 candidate OMIM to DO mappings. This pipeline has identified OMIM IDs (multiple phenotypes and inheritance variants) that represent a single disease that had not previously been identified through DO's UMLS concept mapping or the DO team's curation efforts. For example, 16 additional OMIM IDs were identified, reviewed and integrated into DO for Alzheimer's disease. These OMIM IDs include both ‘Phenotype description, molecular basis known’ (OMIM symbol #) and the ‘Phenotype description, molecular basis unknown’ (OMIM symbol%).

### Assessing disease vocabulary concept overlap

The DO continues to strive to be a resource for unifying disease concepts directly through DO terms and indirectly through DO's extensive cross-mappings. Ultimately, through collaborative development, DO has the goal to provide a complete set of disease term concepts. Evaluation to identify areas of DO to be augmented involves examining concept overlap. As a first step to examine the connectivity of disease vocabularies via cross-references, we carried out a cross-comparison analysis for seven disease vocabularies: the Human DO, National Drug File Reference Terminology (NDF-RT) disease terminology (15), NCI Thesaurus disease terminology, Orphanet (12) Rare Disease Ontology (ORDO, http://www.orphadata.org/cgi-bin/inc/ordo_orphanet.inc.php), Comparative Toxicogenomics Database (CTD) merged disease vocabulary (MEDIC) (16), Kyoto Encyclopedia of Genes and Genomes (KEGG) (17) MEDICUS, and Human Phenotype Ontology (1) disease annotations (HPO-D). These disease vocabularies contain enriched associations between disease terms and other biomedical concepts, including drugs, genes and phenotypes. The means of comparison is via common cross-references. Each of these vocabularies provides cross-references to one or more of MeSH [DO, ORDO, CTD, NDF-RT, KEGG], OMIM [DO, ORDO, CTD, KEGG, HPO-D], SNOMED-CT [DO, ORDO] and UMLS [DO, ORDO, NDF-RT, KEGG, NCI]. Furthermore, HPO-D contains ORDO cross-references, which in turn can be expanded by means of any ORDO cross links to MeSH, OMIM, SNOMED-CT and UMLS to create an indirect set of cross-references from HPO-D to MeSH, OMIM, SNOMED-CT and UMLS.

UMLS includes a meta-thesaurus that combines health and biomedical vocabularies using grouping and sematic integration of synonymous relationships. MeSH, OMIM and SNOMED-CT are among the UMLS source vocabularies; therefore, cross-references to disease concepts from MeSH, OMIM or SNOMED-CT were further (indirectly) mapped to a UMLS disease concept (when such a cross mapping exists within UMLS).

All MeSH, OMIM, SNOMED-CT and UMLS cross-reference links (directly provided by a disease vocabulary or indirectly derived as described above) were used in the comparison analysis. To justify our cross-mapping approach, we have also carried out overlap analysis either without direct links through MeSH, OMIM and SNOMED-CT, or without indirect links through UMLS (see Supplementary Tables S1 and S2). The overlap analysis of cross-references between disease vocabularies changed markedly for practical reasons. For example, using only UMLS for the overlap analysis excluded a number of disease-related OMIM cross-references that did not have a corresponding UMLS concept. Similarly, excluding any indirect UMLS links prevents NCI from having significant overlap with other disease vocabularies, as NCI does not provide any MeSH, OMIM or SNOMED-CT cross-references.

The sizes of the disease vocabularies cross-compared with DO are provided in Table 1 (see also Supplementary Table S3). The NDF-RT ontological framework organizes disease concepts into a hierarchical structure derived from the MeSH vocabulary, and it explicitly asserts different relations between drugs and diseases. NCI Thesaurus integrates different kinds of concept schemes and their interrelationships in a unified conceptual framework. NCI disease terms belong to six semantic types, including disease or syndrome, neoplastic process, pathologic function, mental or behavioral dysfunction, sign or symptom and abnormality. ORDO integrates Orphanet multi-hierarchical classifications of rare diseases with semantic interoperability. KEGG MEDICUS contains integrated information for drugs, diseases and other health-related concepts. CTD MEDIC has been employed to hierarchically annotate disease-associated toxicogenomic relationships and is derived from MeSH and OMIM. MEDIC disease terms were downloaded, and all of the primary/alternative MeSH and OMIM identifiers including the MEDIC Slim identifiers were used in the cross-comparison analysis. HPO-D provides genetic disease annotations for human phenotypic abnormalities using disease concepts in OMIM, Orphanet and DECIPHER databases.

**Table 1.** The seven disease vocabularies being cross-compared.

Vocabulary	Data source	Size	Access date
DO	Subclasses of root disease concept ()	8,757	26 March 2014
NDF-RT	Subclasses of root disease concept (N0000000004)	4,700	26 March 2014
NCI	Subclasses of root disease concept (C2991)	4,252	11 August 2014
ORDO	Subclasses of root disease concept (Orphanet_C001)	4,799	27 March 2014
KEGG	All of KEGG MEDICUS disease concepts	1,359	30 March 2014
CTD	All of MEDIC disease concepts	11,898	08 April 2014
HPO-D	All of hereditary syndromes with phenotypic information	7,520	07 August 2014

### Cross-comparison results and conclusions

The count of unique cross-references provided by each disease vocabulary is summarized in Table 2. UMLS links reported in this table are provided in both ‘indirect’ (i.e. derived via UMLS from MeSH, OMIM and SNOMED-CT links) and ‘direct’ (i.e. provided directly by the vocabulary) form along with the unique count of the union of these two links being ‘combined’, showing there can be substantial unique UMLS cross-references provided using the ‘indirect’ linking method but also strong overlap.

**Table 2.** The number of unique cross-references in each disease vocabulary.

	MeSH	OMIM	SNOMED-CT	UMLS direct	UMLS indirect^a^	UMLS combined	Total
DO	2,908	1,864	13,276	12,170	8,354	13,004	35,895
ORDO	1,705	5,201	1,943	7,997	2,285	8,071	16,920
CTD	11,332	3,977	0	0	6,650	6,650	21,959
NDF-RT	4,661	0	0	4,699	7,922	8,245	12,906
KEGG	1,295	2,690	0	0	4,482	4,482	8,467
HPO-D	0	6,528	0	0	8,000	8,000	14,528
NCI	0	0	0	3,910	0	3,910	3,910
Total^b^	11,467	7,389	14,362	13,486	23,251	23,251	56,469

^a^Inferred UMLS cross-references were obtained by UMLS mapping to MeSH, OMIM and SNOMED-CT concepts.

^b^Total number of unique cross-references.

It is worth noting that DO provides the largest number of unique cross-references (35 895), which contributes ∼64% of the total number of unique cross-references (Table 2). This emphasizes DO's role as a resource rich in cross-references and usefulness as a disease-centric scaffold for data. On average, each DO term has more than four cross-references (35 895/8757). The large number of cross-references indicates the role of the DO in interoperability in the DO domain.

### Mapping methodology

If any two disease terms from different disease vocabularies shared one or more cross-references, identity was assumed and they were treated as synonymous terms. Each vocabulary has its own emphases on particular disease categories. For instance, ORDO and HPO-D are primarily comprised genetic diseases, and NCI Thesaurus has specific focus on cancers. Not surprisingly, ORDO and HPO-D have a high degree of overlap with each other, and other disease vocabularies may have a low degree of overlap with them (Table 3).

**Table 3.** The overlap between disease vocabularies.

	DO	ORDO	CTD	NDF-RT	KEGG	HPO-D	NCI
DO	8,757^a^	1,492	3,432	3,200	1,102	1,278	2,092
ORDO	1,952	4,799^a^	3,503	1,465	2,383	4,170	993
CTD	6,413	6,418	11,898^a^	6,701	6,607	2,883	2,345
NDF-RT	2,878	1,091	4,660	4,700^a^	775	876	1,686
KEGG	854	1,105	1,303	718	1,359^a^	1,102	522
HPO-D	2,291	5,426	3,818	1,169	3,102	7,520^a^	818
NCI	3,692	888	1,004	1,865	625	694	4,252^a^

The number of identical terms between any two disease vocabularies indicates the degree of overlap between them. The off-diagonal numbers in the overlap matrix can be interpreted as the number of terms from the row disease resource covered by the column disease resource. For example, DO has common cross-references with 1492 of the 4799 ORDO terms, while ORDO has common cross-references with 1952 of the 8757 DO terms. The diagonal numbers in the overlap matrix indicate the total number of terms in each disease resource.

^a^The total number of disease terms in each disease vocabulary.

It is noteworthy that most disease vocabularies have very low overlap with NCI Thesaurus except DO. DO has more general coverage of disease categories, but still a relatively low degree of overlap with the rare diseases represented in ORDO and HPO-D, and has moderate overlap with CTD, NDF-RT and KEGG diseases and has high overlap with NCI cancer diseases (Table 3).

The percent overlap between the seven different disease vocabularies is presented in Table 4. There are a number of observations apparent. KEGG has the fewest disease terms and, therefore, a limited ability to cover the disease concepts used by other terminologies; however, it does so rather effectively, covering 56% of CTD and 50% of ORDO. HPO-D extensively uses ORDO to annotate their disease-related terms, so it may not be a surprise that it covers 87% of the ORDO disease terms. For the same reason, it is not very surprising that HPO-D and ORDO both cover about the same amount of the KEGG disease concepts. NCI, with its emphasis on cancer, has limited overlap (at most 38%) with the other disease terminology concepts. NDF-RT's focus on druggable diseases appears to limit its coverage of known diseases without treatments, but it still covers more than half of CTD and KEGG disease concepts. Lastly, it is rather interesting to compare the cross-coverage of CTD and DO. Clearly, CTD covers druggable targets better than DO (as manifest by 99% coverage of NDF-RT) but has limited coverage of cancer disease concepts (as indicated by a 24% overlap with NCI).

**Table 4.**
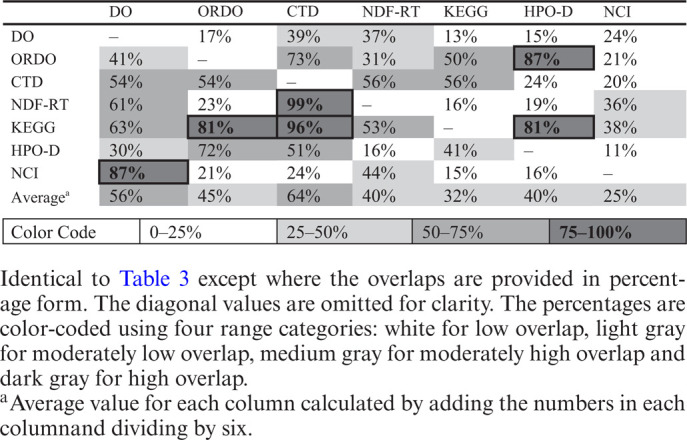
The percentage overlap between disease vocabularies.

Alternatively, DO has good coverage of cancer concepts (as indicated by 87% overlap with NCI) but has only moderate coverage of druggable/treatable diseases (as indicated by a 61% overlap with NDF-RT). This indicates areas of strength and improvement for DO to cover additional disease concepts (e.g. by identifying missing NDF-RT disease concepts).

### Updates to the DO website

The current version of the DO website (7) (version 1.0) provides a comprehensive resource to perform full-text searching on the DO as well as exploring and visualizing relationships between terms. The DO database website has been updated periodically since 2012 to include major data releases. The DO website provides links to DO related resources (DO tracker, downloading the HumanDO.obo file and DO tutorial). The Resources Tab has been augmented with links to disease—gene resources (FunDO (18), Bioconductor GeneAnswers (19)) and downloads of the DO Neo4j database on GitHub. The DO API allows for querying of terms for a specific DOID through a REST-style URL. For example, the URL for DOID:11725 is http://www.disease-ontology.org/term/DOID:11725/.

### DO site usage and mappings statistics

Following DO's 2012 NAR paper, the number of sessions and users (Google Analytics) on the DO website has risen dramatically, with the number of returning visitors rising from 18 to 58% between December 2011 and June 2014 (Table 5).

**Table 5.** Google analytics reporting for the DO website.

Time frame	Sessions	Users	Returning visitors	New visitors
8/31/2013–9/1/2014	11,517 (total)	6,206	47%	53%
December 2011	539 (per month)	446	18%	82%
June 2014	1,385 (per month)	681	58%	42%

The increased number of visits (sessions), number of users and percent of returning visitors to the DO website over the past year and monthly averages between December 2011 and June 2014.

The extent of DO's ontology mappings between BioPortal ontologies demonstrates the extended of usage of DO as a disease domain ontology. As of 15 September 2014, BioPortal has mapped DO classes to classes within 128 other BioPortal ontologies (http://bioportal.bioontology.org/ontologies/DOID/?p=mappings).

## DO AVAILABILITY

The DO Neo4j database, HumanDO.obo and HumanDO.owl files are available under the Creative Commons Attribution 3.0 Unported License (http://creativecommons.org/licenses/by/3.0/) which allows for the copying, redistribution and adaption of the ontology for any purpose. The DO files are available in both OBO and OWL format from DO's SourceForge site (http://sourceforge.net/p/diseaseontology/code/HEAD/tree/trunk) and can be found at http://purl.obolibrary.org/obo/doid.obo and http://purl.obolibrary.org/obo/doid.owl. DO's Neo4j database is on GitHub (http://github.com/IGS/disease-ontology). DO's OBO and OWL files are also available from the OBO Foundry (http://www.obofoundry.org/cgi-bin/detail.cgi?id=disease_ontology) and GitHub (http://github.com/obophenotype/human-disease-ontology/HumanDO.owl).

### Community feedback and data submissions

The DO team receives community feedback on a continual basis through individual new term requests, requests for definition, synonym or term updates and requests for explanations of the DO curatorial process and curation decisions. Requests are received through multiple methods including DO's SVN term tracker (http://sourceforge.net/p/diseaseontology/feature-requests/), DO's Contact Us (http://www.disease-ontology.org/contact/), through DO's website feature ‘Add an item to the term tracker’ found at the bottom of each metadata page for each DO term, and direct emails to the DO PIs. The *DO listserv* (diseaseontology-discussion) has received over 200 submissions since November 2011. The DO team has fielded 106 distinct postings through the DO website (DO disease-ontology.org requests) and 59 feature requests through the DO SVN site. Each review request involves examination of the current disease information in DO and examination of current literature and online expert resources (e.g. GeneReviews (20), Orphanet, OMIM, NIH Institutes and MayoClinic) to identify the most appropriate classification for each disease term. The DO team provides prompt replies to each user request at the conclusion of the curation.

Examples of the most often types of requests include: refining DO textual definitions, creating new DO classes (e.g. 25 new DO terms for the fission yeast database), adding DO terms for a set of diseases (e.g. dystonia diseases), term name fixes, term status (obsolete), adding comments to obsolete DO records, adding synonyms from publications to a DO term, removing term redundancy among the synonyms, adding apostrophe free synonyms to disease terms, identifying typos in term names or definitions, adding OMIM IDs to specific DOIDs, updating term parentage or adding relations to definitions to further clarify etiology.

### Large-scale community data submissions

We report here DO's collaborative efforts with biomedical resources over the past two years. DO provides individual curation efforts to coordinate, map and integrate the disease terms used by each biomedical resource. DO works directly with community members providing disease curation to support disease representation among the Model Organism Databases (FlyBase (Susan Tweedie), WormBase (Ranjana Kishore), PomBase (Antonia Lock), within pathway (Reactome: Peter D'Eustachio) and epitope (Immune Epitope Database (IEDB), Bjorn Peters IEDB Disease Finder, http://www.iedb.org/home.php) databases and to foster the development of gene—variant—phenotype resources (e.g. Gene Wiki (21), OMIM API) and cancer variant projects (e.g. The Jackson Laboratory for Genomic Medicine (http://www.jax.org/ct/), HIVE (22)). The Biomuta–HIVE project is ongoing. The DO team is collaboratively building a DO_Cancer_Slim (366 terms mapped to 70 term DO Cancer Slim) derived from cancer terms from: COSMIC, UniProt, TCGA, IntOGen, ICGC and publications. The DO Cancer Slim collaborators include the HIVE team, COSMIC, Novartis and EDRN. The Project Leadership Team includes Raja Mazumder, Tsung-Jung Wu and Krista Smith (George Washington University).

### Biomedical resources incorporating DO

DO terms and IDs are being used to develop and build algorithms, computational tools and biomedical resources. DO has been incorporated into a growing number of biomedical resources including the EBI Array Express (23), the Neuroscience Information Network (NIFSTD) (24), Neurocarta (25), NeuroDevNet (26), Infectious Disease Ontology (http://infectiousdiseaseontology.org), the MIxS genomic metadata standard (27) and the NIH Library of Integrated Network-based Cellular Signatures program (28).

DO has provided disease mappings in the past year across a number of biomedical resources (Table 6). Additionally, the DO team has ongoing interactions with Mouse Genome Informatics (MGI) (mapping OMIM to DO), dictyBase, EMBl_EBI Samples, Phenotypes and Ontology team, the HPO, OMIM (omim.org), Orphanet, PubChem, the Sifem Inner Ear disease project (http://www.sifem-project.eu), cognitive atlas (http://www.cognitiveatlas.org): a knowledge base for mental function, the Protein Ontology (PRO) (http://pir.georgetown.edu/pro/pro.shtml), the Monarch Diseases and Phenotype project (http://monarchinitiative.org) and Gene Wiki.

**Table 6.** The number of DO terms mapped to biomedical resources.

Biomedical resource	Data types	DO disease mappings
Reactome	Disease to pathway	770+
Neurocarta	Disease to gene associations	1,920
FlyBase	Human disease models - Drosophila alleles	2,289
PRO	Protein-disease	50
EFO	EFO-DO	137
NeuroDevNet	Intellectual disabilities	71
	Inherited metabolic disorders	80

DO has become a disease knowledge resource for the further exploration of biomedical data including measuring disease similarity based on functional associations between genes (29), as a disease data source for the building of biomedical databases, e.g. cdGO: an ontology database for protein domains (http://supfam.org/SUPERFAMILY/dcGO) (30) and defining disease-gene relationships, e.g. DGA: Disease and Gene annotations resource (31).

## FUTURE DIRECTIONS

The DO team recognized the imperative need to provide definitions for all DO terms. Integration of textual definitions for all DO terms is a major curatorial effort of the DO team in the next year. DO is moving to a multi-editor curation model in the fall of 2014 in order to improve DO's interoperability, to enable integration of cross products within DO and to develop inferred DO hierarchies (genetic, clinical) in addition to DO's asserted etiology-based hierarchy. This work will involve: moving curation effort to Protégé (coordinated by Chris Mungall) to use OWL and reasoning; creating cross product terms for better interoperability with the OBO Foundry ontologies; and engaging community partners (EBI, MGI, HPO and Orphanet) and cardiovascular and metabolic disease clinicians to join DO as editors and data reviewers. An initial list of the types of data that will be added to DO, along with their associated relations, has been proposed and will undergo additional review within the DO group and among community ontology partners including Barry Smith. The data types, source ontologies and relations to be added to DO include: phenotypes (HPO/PATO: has_phenotype), symptoms (SYMP: has_symptom), age of onset (HPO), anatomical location (UBERON: located_in), GO annotations (Gene Ontology: disregulated_in), cells/tissues of origin (Cell ontology: derives_from or has_material_basis_in) and types of inheritance (has_physical_basis_in). For example, the DO term inhalation anthrax is currently defined as ‘is_a anthrax disease’. The addition of UBERON (32) cross products will utilize the located_in relation to connect the DO term to the UBERON terms lung (UBERON:0002048) and lymph node (UBERON:0000029).

### DO website: future directions

Additional development of the DO website is planned over the next year. Version 2.0 of the DO website will include bulk querying and API development, saving of queries and result datasets and better direct-link support to DO terms. An enhanced DO API will allow users to perform any action that they can do interactively on the website via the API. This will include searching using all fields provided, pulling down static images of visualized term relationships and pulling down single or many sets of term metadata. Expansion of the API will provide another robust resource to allow tech-savvy users to scrape or pull down large amounts of metadata for use in their own websites, web applications or bulk analysis.

## SUPPLEMENTARY DATA

Supplementary Data are available at NAR Online.
